# Recent advances in understanding prodrug transport through the SLC15 family of proton-coupled transporters

**DOI:** 10.1042/BST20180302

**Published:** 2020-03-27

**Authors:** Gurdeep S. Minhas, Simon Newstead

**Affiliations:** Department of Biochemistry, University of Oxford, Oxford OX1 3QU, U.K.

**Keywords:** drug transport, membrane transport, prodrug recognition, SLC15

## Abstract

Solute carrier (SLC) transporters play important roles in regulating the movement of small molecules and ions across cellular membranes. In mammals, they play an important role in regulating the uptake of nutrients and vitamins from the diet, and in controlling the distribution of their metabolic intermediates within the cell. Several SLC families also play an important role in drug transport and strategies are being developed to hijack SLC transporters to control and regulate drug transport within the body. Through the addition of amino acid and peptide moieties several novel antiviral and anticancer agents have been developed that hijack the proton-coupled oligopeptide transporters, PepT1 (SCL15A1) and PepT2 (SLC15A2), for improved intestinal absorption and renal retention in the body. A major goal is to understand the rationale behind these successes and expand the library of prodrug molecules that utilise SLC transporters. Recent co-crystal structures of prokaryotic homologues of the human PepT1 and PepT2 transporters have shed important new insights into the mechanism of prodrug recognition. Here, I will review recent developments in our understanding of ligand recognition and binding promiscuity within the SLC15 family, and discuss current models for prodrug recognition.

## Introduction

**Figure 1.**
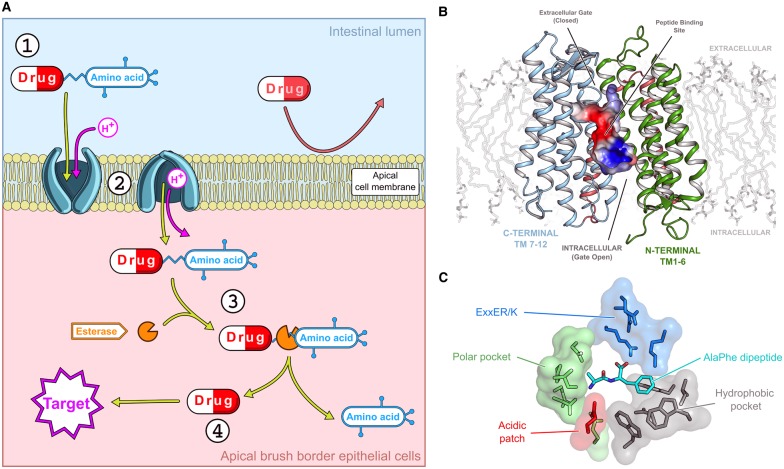
Peptide transporters are targeted to improve drug transport into the body. (**A**) The addition of an amino acid to a drug molecule results in the generation of a prodrug (1) that is able to utilise the intestinal proton-coupled peptide transporter, PepT1, for active transport across the cell membrane and into the body (2). Once in the cytoplasm, the prodrug is acted upon by enzymes that cleave the linker bond (3) and release the parent drug (4). (**B**) Crystal structure of a POT family transporter showing the N- and C-terminal bundles, coloured green and blue, respectively. The central peptide binding site is shown in surface electrostatics (blue positive; red negative). The transporter is shown in the inward-open conformation, with the peptide binding site closed to the extracellular side of the membrane and open to the cytoplasm. Two gates control access to the binding site, which alternately open and close in response to peptide and proton binding. (**C**) The peptide binding site contains several specificity pockets that recognise peptides and their associated side chains.

Solute carrier (SLC) transporters are important determinants of drug pharmacokinetics and are increasingly being identified as important therapeutic targets in their own right [1,2]. Poor oral bioavailability is one of the leading causes of failure in preclinical and clinical drug development and is viewed as a major challenge in the pharmaceutical and biotechnology industry [3]. One approach to address this challenge has been the development of prodrugs that target the intestinal peptide transporter, PepT1 (SLC15A1), which is highly expressed in the brush border membrane of the small intestine [4,5] (Figure 1A). The central idea is to modify existing drug molecules so that they resemble the physiological peptides found in the small intestine and thereby co-opt the peptide transporters into driving drug their uptake into the body. Modifying drugs to improve their pharmacokinetic properties results in so-called prodrugs, which are generally bioreversible derivatives of the parent drug molecules that undergo an enzymatic or chemical transformation *in vivo* to release the active parent drug [6]. Over the past 10 years, significant effort has been made in the design of novel prodrug molecules with improved pharmacokinetic profiles [7,8]. One successful approach has been to use amino acids as promoieties, as these confer several advantages on the parent compound, including increased water solubility and the targeting of intestinal SLC transporters for oral drug delivery [9].

However, targeting specific SLC transporters for carrier mediated uptake is still a major challenge and made more difficult due to the absence of structural and biochemical information on many SLC transporters [10]. Two members of the SLC15 family of proton-coupled peptide transporters, PepT1 (SLC15A1) and PepT2 (SLC15A2) exhibit remarkable ligand promiscuity, and are known to transport many different drug molecules, including angiotensin-converting enzyme inhibitors, beta-lactam antibiotics, an *N*-methyl-d-aspartate receptor antagonist PD-15874 and 5-aminolevulinic acid, currently being evaluated as a treatment for bladder and oesophageal cancer [11–13]. In a quirk of physiological fate, these transporters perform a virtuous cycle if targeted correctly, with PepT1 being expressed in the small intestine and so able to effectively transport drugs into the blood stream, and PepT2 expressed in the kidney nephrons, acting to retain the drugs and exclude their excretion in the urine. Additionally, PepT2 is also expressed in the problematic blood-brain barrier, where it functions to control peptide transport into the central nervous system [14]. It is perhaps not surprising, therefore, that prodrugs targeting both PepT1 and PepT2 show favourable absorption and retention profiles in animal models of drug disposition and are being actively pursued as valid targets for improving biopharmaceutical and pharmacokinetic profiles in humans [15–17].

A major breakthrough in carrier mediated prodrug development was the introduction of the antiviral valacyclovir in 1995, marketed under the trade names Valtrex and Zelitrex. Valacyclovir is a prodrug derivative of the antiviral agent acyclovir, which is used in the treatment of herpes virus as well as in prophylaxis against the acquisition of infection and in the suppression of latent disease [18]. This was soon followed by another prodrug, valganciclovir in 2001, which is used to treat cytomegalovirus infections in patients with HIV/AIDS or following organ transplant [19] and marketed under the trade name Valcyte. The oral bioavailability of both valacyclovir and valganciclovir improved to >50% for the prodrug derivative, which was attributed to its recognition and transport by intestinal peptide transporter PepT1 [20–24]. Intestinal transporters are often targeted for prodrug transport, as they function to rapidly transport a variety of chemical different molecules into the blood stream from the diet [8]. Within the repertoire of intestinal SLC transporters, PepT1 stands out as displaying the most extreme promiscuity [25]. This characteristic, coupled with the similarity of both amino acid side chains to functional groups of drug molecules and the size of di- and tri-peptides to small molecule therapeutics [11], has resulted in PepT1 being a major focus of prodrug design strategies [26–28]. However, the lack of a high-resolution 3D structure and associated pharmacophore model for drug binding has hampered progress [29]. Recently, however, three co-crystal structures have been reported that show two different bacterial peptide transporters in complex with two prodrug molecules and the peptide-based photodynamic therapy agent, 5-aminolevulinc acid [30,31]. Bacterial peptide transporters have proven to be valid model systems with which to understand the molecular basis of peptide recognition within the human transporters [32–34]. In the context of several previous structural studies showing how this family of transporters recognise and transport physiological peptides across the membrane, a picture now starts to emerge of a unified binding mechanism that will likely form the basis for developing a more accurate pharmacophore model for drug recognition and transport within the human SLC15 family [35].

## SLC15 transporters belong to the larger POT family of proton-coupled peptide transporters

PepT1 and PepT2 belong to the much larger POT or PTR family of proton-coupled oligopeptide transporters, with homologues found in all domains of life except the archaea [36,37]. POT family transporters belong to the major facilitator superfamily (MFS) of secondary active transporters, and use the proton electrochemical gradient to drive the concentrative uptake of di- and tri-peptides into the cell [38]. Structurally they contain 12 transmembrane (TM) spanning alpha helices, which pack together into two six-helix bundles in the membrane [39] (Figure 1B). A central ligand-binding site is located within the centre of the molecule, flanked on either side by the two six-helix bundles. Access to the central binding site is controlled by two gates at the extracellular and intracellular end of the central cavity [40]. The gates themselves are made up of four pairs of helices, two from each of the six-helix bundles [41]. Several studies have determined that the gates are opened and closed in response to both peptide and proton binding, which appear to regulate the formation and breakage of conserved salt bridge interactions made between the gating helices [42–45]. A remarkable feature of POT family transporters is the promiscuity of the binding site, with the upper estimate for the number of di and tri-peptide ligands that can be recognised and transported in the several thousand [46]. Here structural studies have built on previous electrophysiological and biochemical transport studies to reveal a highly charged binding site that exhibits a pronounced dipolar character (Figure 1C) [4,40]. Crystal structures and biochemical assays have further revealed a general model for how peptides are recognised [34,47–49], with important roles for conserved hydrophobic and polar pockets. In brief, the amino and carboxy termini of the peptides are recognised by conserved acidic and basic side chains within the binding site, with specificity provided by pockets with varying degrees of polar and hydrophobic character, which accommodate the different side chains. Additionally, several POT transporters also appear to recognise peptides in different orientations, with larger tri-peptides accommodated in vertical orientations and smaller di-peptides being held in a more horizontal orientation [47,50]. Interested readers are directed to towards recent reviews that discuss these results and the mechanisms for proton-coupled peptide transport [33,38]. Here, we wish to focus instead on how two recent structures of prodrug complexes have expanded our understanding of drug recognition and pharmacophore development.

## Rationalising prodrug recognition

**Figure 2.**
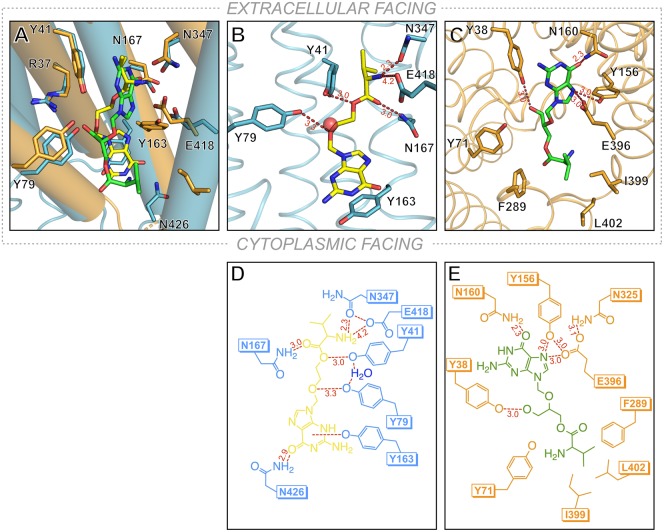
Comparison of the crystal structure of POT transporters in complex with prodrugs valacyclovir and valganciclovir. (**A**) Overlay of the two prodrugs in their respective transporters. Valacyclovir (yellow) was captured in PepT_Sh_ (blue), while valganciclovir (green) was captured in DtpA (brown). Amino acid positions are labelled for PepT_Sh_. (**B**) View of the binding site for valacyclovir in PepT_Sh_ (PDB: 6GZ9). (**C**) View of the binding site for valganciclovir in DtpA (PDB: 6GS4). (**D**) Schematic view of the binding interactions for valacyclovir. (**E**) Schematic view of the binding interactions for valganciclovir. Hydrogen bonds are shown in dashed red lines, distances shown are in Ångstroms.

Recently two crystal structures of different POT family transporters in complex with the antiviral prodrugs valacyclovir and valganciclovir were reported [30,31]. These two prodrugs are very similar, except for the presence of a methoxy group in the ganciclovir parent molecule and both are current prescribed to treat cytomegalovirus infections. The crystal structures revealed the two molecules in the central peptide binding site, but interestingly in different orientations (Figure 2A). In the valacyclovir complex, which was captured in the POT family transporter from *Staphylococcus hominis*, PepT_Sh_, the prodrug molecule is seen with the valine moiety orientated towards the extracellular gate, with the amino terminus making both a hydrogen bond and salt bridge interaction with Asn347 and Glu418 (PDB: 6GZ9) (Figure 2B). The latter residue being an important component of the proton coupling mechanism [43]. The ester group, which connects the valine amino acid to the parent drug molecule, interacts via another hydrogen bond to one of two conserved tyrosine side chains, Tyr41, which was identified as playing an important role in ligand specificity [43]. Valacyclovir also contains an ether bond, which belongs to the parent drug molecule, which also interacts with a conserved tyrosine, Tyr79, again identified as playing an important role in ligand specificity within the wider SLC15/POT family [38]. Of particular interest is how the parent drug molecule is accommodated. As mentioned, in the peptide complexes we have observed that large side chains are accommodated in specificity pockets. Indeed, we suggested a similar situation may occur for prodrug molecules, with the drug component being accommodated within the hydrophobic pocket we observed previously [51]. However, in this structure, the acyclovir component is accommodated through a pi–pi stacking interaction with another tyrosine, Tyr163. Interestingly this tyrosine forms part of the PTR2_2 (FYxxINxG) motif on TM4 in PepT_Sh_, which forms part of the intracellular gate in POT family transporters. The function of the PTR2_2 motif is currently unclear, but it is highly conserved in the bacterial POT family transporters [36] and the equivalent tyrosine in rabbit PepT1 is essential for transport [52], suggesting it may play a similarly important role in drug transport in the human proteins.

The second structure of a prodrug complex was reported DtpA, from *Escherichia coli*, in complex with valganciclovir (PDB: 6GS4) [31]. Although very similar to valacyclovir, the valganciclovir molecule sits in a different orientation, flipped almost 180° relative to valacyclovir (Figure 2A,C). In this structure, it is the nucleoside analogue that interacts with the conserved glutamate on TM10, Glu396 in this protein, via amid nitrogen N16 (Figure 2E). The conserved asparagine on TM 8, Asn325, that was observed interacting with the amino group in valacyclovir, is not interacting with the prodrug in DtpA, instead this side chain makes an interaction to Glu396. Similarly, to the valacyclovir structure, the carbonyl group on the nucleoside moiety interacts with another conserved asparagine, Asn 160 on TM4. However, in the PepT_Sh_ structure, the asparagine is different, and on TM10. Also dissimilar to the valacyclovir structure, the hydrogen bond interactions with valganciclovir cease at the additional methoxy group of the parent drug, which interacts with a conserved tyrosine on TM1, Tyr38. The remaining valine amino acid is accommodated in a hydrophobic pocket formed by Try71, Phe289, Ily399 and Leu402.

**Figure 3.**
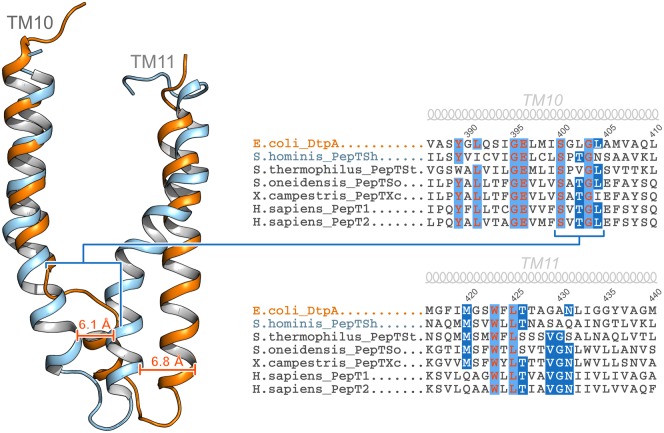
Helix 10 in DtpA adopts an usual conformation compared with PepTSh. Structural comparison of the TM10 and TM11 helices in DtpA and PepT_Sh_. Helix 10 in DtpA adopts an unwound conformation towards the cytoplasmic end of the helix, resulting in a pronounced displacement of the backbone relative to PepT_Sh_. A sequence alignment of DtpA with PepT_Sh_ and other bacterial POT family transporters is shown, revealing that the structural difference cannot be predicted from the primary structure.

What could account for this difference in binding orientation? Interestingly in the DtpA structure, the region around TM10 is structurally very different when compared with PepT_Sh_ (Figure 3). In DtpA the region towards the cytoplasmic end of the helix forms a disordered region, before reforming the helix and connecting into TM11. In comparison, in PepT_Sh_ TM10 forms a single helical structure, with no similar disordered loop. Given the importance that TM10 and 11 play in regulating access to the central peptide binding site during transport [41], it is highly likely this structural difference will impact how these proteins recognise peptides and prodrugs. Indeed, substrate preferences between di- and tri-peptides are much more pronounced in bacterial members of the POT family, with reports that the *E. coli* YjdL transporter preferring di-peptides over larger tri-peptides for example [53]. It is possible that similar differences in helix structure within the different POT family transporters have resulted in altered substrate preferences and peptide binding orientations. Unfortunately, it appears that sequence alone is insufficient to determine whether this structural characteristic is present in the mammalian transporters, as the sequence of the proteins in this region is very similar between DtpA and PepT_Sh_.

## An emerging pharmacophore and peptide binding model

**Figure 4.**
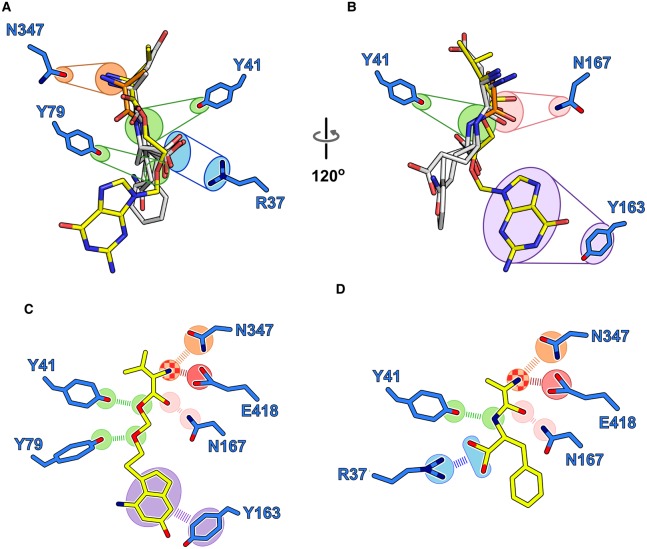
Valacyclovir adopts a similar binding position to physiological peptide ligands. (**A**) Structural overlay of the valacyclovir structure onto PepT_St_ (4D2B) and in complex with peptides Ala-Phe (PDB:4D2C), Ala-Gln (5OXK), Asp-Glu (5OXM), and tri-alanine (PDB:4D2D). Common points of contact are highlighted. (**B**) View rotated 90° relative to (**A**), showing the involvement of the PTR2_2 tyrosine.

A key aim of research into the POT family of peptide transporters is to gain a working understanding of how these proteins recognise and transport drug molecules across the cell membrane. An accurate pharmacophore model, supported by cell and biochemical transport assays has been a key goal of the transport field [35]. After several years of effort from multiple researchers around the world, we now have a selection of high-quality crystal structures of POT family transporters in complex with different physiological peptide ligands (Table 1). These include six complexes with di-peptides and three with tri-peptides. Given the reasonable sequence identity between the mammalian and bacterial proteins, we can use these structures to gain important insights into how the mammalian peptide transporters might recognise peptides and drug molecules in the human body. Fortuitously all of the current crystal structures have been captured in essentially the same inward-facing conformation, with the binding site open to the cytoplasm and sealed on the outside of the cell. This enables us to superimpose the structures and observe any similarities in how these different peptide transporters interact with their ligands. Indeed, we observe several commonalities in the way these proteins recognise their peptide ligands and the prodrug valacyclovir (Figure 4A,B). It appears the amino terminus of the peptides and valine moiety of the prodrug interact with a conserved asparagine on TM10, Asn347. Interestingly, the carboxy termini of the peptides also cluster in one region of the binding site, interacting with the two conserved tyrosines on TM1 and 2, Tyr41 and 79, respectively. In the valacyclovir complex, it is these tyrosines that interact with the ester and ether bonds of the prodrug (Figure 2B,D). Tyrosines are well known to play important roles in promiscuous binding sites in proteins, and are often concentrated in the CDR3 loop of antibodies [54]. It is likely that they play a similar role in increasing the repertoire of interactions available within the binding site of POT family transporters. Supporting this hypothesis, we observe the acyclovir drug group, which is a nucleoside analogue, accommodated through a pi–pi stacking interacting with another tyrosine, Tyr163 on TM4. The last common interaction point we observe is with the carbonyl group, which makes a conserved interaction with Asn167, also on TM4.

**Table 1.** Crystal structures of all currently reported POT family peptide transporters

POT transporter	Ligand	Conformation	PDB	Literature
PepT_So2_ (*Shewanella oneidensis*)				
	*apo*	Inward-open	2XUT	Newstead et al. [40]
	*apo*	Occluded state	4UVM	Fowler et al. [41]
	Alafosfalin	Inward-open	4LEP	Guettou et al. [56]
	Ala-Tyr	Inward-open	4TPH	Guettou et al. [34]
	Ala-Ala-Ala	Inward-open	4TPJ	
	Ala-Tyr-Ala	Inward-open	4TPG	
	*apo*	Inward-open	6JI1	Nagamara et al. [57]
PepT_St_ (*Streptococcus thermophilus*)				
	*apo*	Inward-open	4APS	Solcan et al. [43]
	*apo*	Inward-open	4XNJ	Huang et al. [58]
	Ala-Phe	Inward-open	4D2C	Lyons et al. [47]
	Ala-Ala-Ala	Inward-open	4D2D	
	Ala-Phe	Inward-open	5D58	Huang et al. [59]
	*apo*	Inward-open	5OXO	Martinez-Molledo et al. [49]
	*apo —* PO_4_ bound	Occluded state	5OXP	
	Ala-Leu	Inward-open	5OXL	
	Ala-Gln	Inward-open	5OXK	
	Asp-Glu	Inward-open	5OXM	
	Phe-Ala	Inward-open	5OXN	
	Phe-Ala-Gln	Inward-open	6GHJ	Martinez-Molledo et al. [49]
	HEPES	Inward-open	6EIA	
PepT_Sh_ (*Staphylococcus hominis*)				
	Cys-Gly-3M3SH	Inward-open	6EXS	Minhas et al. [50]
	Valacyclovir	Inward-open	6GZ9	Minhas and Newstead [30]
	5-aminolevulinic acid	Inward-open	6HZP	
GkPOT (*Geobacillus kaustophilus*)				
	*apo*	Inward-open	4IKV	Doki et al. [48]
	*apo —* E310Q	Inward-open	4IKX	
	*apo —* E310Q SO_4_ bound	Inward-open	4IKY	
	SO_4_	Inward-open	4IKW	
	Alafosfalin	Inward-open	4IKZ	
DtpA (*Escherichia coli*)				
	Valganciclovir	Inward-open	6GS4	Ural-Blimke et al. [31]
	MES	Inward-open	6GS1	
	Glycine	Inward-open	6GS7	
YbgH (*Escherichia coli*)				
	*apo*	Inward-open	4Q65	Zhao et al. [60]
PepT (*Yersinia enterocolitica*)				
	*apo*	Inward-open	4W6V	Boggavarapu et al. [61]
PepT_Xc_ (*Xanthomonas campestris*)				
	*auto-inhibited*	Inward-open	6EI3	Parker et al. [42]

Taken together, we propose an initial binding model for how SLC15 family transporters interact with amino acid-based prodrug molecules (Figure 4C,D). Based on the crystal structures of POT family transporters, we proposed that peptides interact primarily through their amino and carboxy termini, which make salt bridge interactions to the conserved glutamate on TM10 and arginine on TM1, respectively. Our valacyclovir structure reveals that the amino terminus of the valine moiety of the prodrug does indeed interact with the conserved glutamate, however, we did not observe an interaction with the arginine on TM1, possibly due to the absence of a carboxy group in the prodrug, or maybe steric restrictions resulting from accommodating the larger drug group in the binding site. However, we did observe interactions conserved interactions with a tyrosine on TM1, which recognises the peptide bond in peptides and the ester group in valacyclovir, and an asparagine on TM4, which recognises the carbonyl group of the peptide bond in peptides and the equivalent carbonyl group in valacyclovir. In many ways, this model supports earlier suggestions that addition of the amino acid to drug molecules enables the prodrug to dock into the binding site, whereupon the promiscuous nature of the site, generated from the increased concentration of tyrosine side chains, accommodates the remaining drug group [51]. This model also sits in good agreement with an earlier binding model generated for the rabbit peptide transporter, PepT1, which also noted the importance of the interactions to the amino group of the peptide to the overall affinity of the ligands for the transporter [4,55].

This model, however, is difficult to reconcile with the valganciclovir structure obtained for DtpA, which as discussed above adopts a very different orientation in this transporter. However, we have seen in other POT family transporters that part of the mechanism by which these proteins accommodate such a large repertoire of ligands is through the use of multiple binding modes within the same site [47,49]. It may well be the case that slight changes to the drug molecules enable a different binding orientation, or that changes in the structure of the binding site, as observed in TM10 in DtpA, enable different sets of ligands to be accommodated. It should be remembered that peptide transporters function to transport peptides en masse into the cell; they have evolved as bulk carriers, not selective uptake systems. Specificity has been sacrificed for efficiency and promiscuity. The different binding orientations observed between valacyclovir and valganciclovir may be indicating that multiple pharmacophore models exist for PepT1 and PepT2.

## Future questions

The era of precision medicine is rapidly approaching, with advances in genomics and metabolomics enabling a much greater understanding of how human beings metabolise and respond to different drug regimens. Understanding the role and regulation of SLC transporters in the human body is a major plank of this worldwide endeavour. The ability to co-opt intestinal and renal drug transporters to improve drug absorption and retention has been a success to date with antivirals and antineoplastic agents, but is ultimately hampered by a lack of high-resolution structural, biochemical and biophysical data on how these systems interact with drug molecules. The structures presented here sound the starting gun towards a unified pharmacophore model to understand how best to target PepT1 and PepT2 for improved drug delivery and reduced cytotoxicity in the human body.

## Perspectives

- SLC proteins are responsible for transporting drugs into and throughout the human body; they play a major role in drug pharmacokinetics.
- The SLC15 family of peptide transporters have been targeted to improve the oral bioavailability and renal retention of antiviral prodrugs valacyclovir and valganciclovir.
- Recent crystal structures of bacterial SLC15 homologues in complex with antiviral prodrugs have revealed how these two drug molecules are recognised, paving the way for more targeted prodrug design.

## Abbreviations

MFS: major facilitator superfamily
SLC: solute carrier
TM: transmembrane

## Open Access

Open access for this article was enabled by the participation of University of Oxford in an all-inclusive *Read & Publish* pilot with Portland Press and the Biochemical Society under a transformative agreement with JISC.
